# Prevalence of recurrent aphthous ulceration in the Indian Population

**DOI:** 10.4317/jced.51227

**Published:** 2014-02-01

**Authors:** Santosh Patil, S N. Reddy, Sneha Maheshwari, Suneet Khandelwal, D. Shruthi, Bharati Doni

**Affiliations:** 1 Department of Oral Medicine and Radiology, Jodhpur Dental College, Jodhpur National University, Jodhpur (Raj). India; 2 Department of Oral Medicine and Radiology, Desh Bhagat Dental College, Muktsar (Punjab). India; 3 Department of Oral and Maxillofacial Pathology, Desh Bhagat Dental College, Muktsar (Punjab). India; 4 Department of Public Health Dentistry, Pananeeya Institute Of Dental Sciences, Hospital and Post Graduate Research Centre, Hyderabad (Andhra Pradesh). India; 5 Department of Oral Medicine and Radiology, NIMS University Dental College, Jaipur (Raj). India

## Abstract

Objective: Patients with an oral ulcer may present initially to a general physician or a dental practitioner. Majority of the ulcers are benign and resolve spontaneously but small proportions are malignant. The aim of the present study was to determine the prevalence of recurrent aphthous ulcerations in the Indian population. Material and Methods: 3244 patients attending the Department of Oral Medicine and Radiology during the period from November, 2010 to December, 2012 with various complaints were examined. Of the patients examined 1669 were females and 1575 were males. Results: 705 patients presented with recurrent aphthous ulceration (21.7%). Females (56.3%) were more commonly affected than males (43.7%). Patients in the third and fourth decade were most commonly affected. Stress was the most common factor associated with recurrent aphthous stomatitis (386 patients). 54.5% patients did not take any medications and 72.9% patients opined that the condition needed no dental consultation. Conclusion: The results of the present study indicate that recurrent aphthous ulceration is a common mucosal disorder in the Indian population. The early and proper diagnosis of the ulcers will help the dental practitioner in providing information to the patient regarding awareness and management of the condition.

** Key words:**Recurrent aphthous ulcers, prevalence, Indian population.

## Introduction

Oral ulceration is a common complaint of patients attending out-patient department. The estimated point prevalence of oral ulcers worldwide is 4%, with aphthous ulcers being the most common, affecting as many as 25% of the population worldwide (1). The term “aphthous” is derived from a Greek word “aphthae” which means ulceration (2). The term was first used by Hippocrates and later described by Mikulicz and Kummel as ‘Mikulicz’s aphthae’ (3). These present clinically as multiple, small, round, or ovoid ulcers, with circumscribed margins, covered by a yellowish or gray-white fibrinous exudate and surrounded by an erythematous halo, and present first in childhood or adolescence (2). There is intense or moderate pain and the ulcers heal in 10-14 days for the more common type and more than 2 weeks for the severe type. Recurrence of the ulcers occurs in intervals within a year or over several years (4).

There are many causes of oral ulcerations, which may overlap, and thus the etiology becomes unclear, as observed in recurrent aphthous stomatitis (RAS). The etiology of recurrent aphthous stomatitis is uncertain, and both environmental and genetic factors are indicated. It has been associated with a number of causes including stress, trauma, infection, allergy, genetic predisposition, or nutritional deficiencies (5,6). None of these etiologies has been validated. Since, these ulcers have a significant negative impact on the oral health, irrespective of the cause, affecting the quality of life; it is important to know the prevalence of these ulcerations in the general population.

No studies have been done till date to estimate the prevalence of RAS in the Indian population. The present study was designed to find out the prevalence of recurrent aphthous stomatitis among the Indian population and assess the independent factors related to this oral mucosal condition.

## Material and Methods

A prospective study was carried out in the Department of Oral Medicine and Radiology of Jodhpur Dental College General Hospital during the period from November, 2010 to December, 2012. 3244 patients with various complaints were examined. Ethical clearance from the Institutional Ethical Committee was obtained. A written informed consent was obtained from the patient. Of the patients examined 1669 were females and 1575 were males. Patients of all age groups attending the routine dental checkup were examined for RAS. All patients with suspected malignant ulcers based on clinical examination were excluded from the study. The clinical examination of the oral cavity was done following the WHO guidelines (4), under artificial illumination on a dental chair, using a mouth mirror. The aphthous included the minor, major and herpitiform types, which are characterized by recurrence. A detailed family and medical history and history in relation with any habits of tobacco/smoking/alcohol was recorded. Medical history included any systemic disease, association of ulcers with menstrual cycle, any medication, and any associated ulcers involving organs like skin, vagina, eye or joints. The patients were asked if any medication was taken to treat the condition or relieve the symptoms. Patients were also enquired about other independent factors related to the condition. Lymph nodes were also examined. For all patients hematological investigations that included Full blood count (FBC), erythrocyte sedimentation rate (ESR), Rheumatoid factor, serum folate, vitamin B12 and ferritin levels, were advised. The observations were entered and analyzed using the computer program, SPSS 12 (SPSS Inc. Chicago, USA).

## Results

705 patients of the total 3244 patients presented with recurrent aphthous ulcers at the time of examination, giving an overall prevalence of 21.7% ( Table 1). Patients in the third (20.7%) and fourth (26.5%) decade were most commonly affected. Females (56.3%) were more commonly affected than males (43.7%) ( Table 2) and the gender difference was statistically significant (p<0.005).  Table 3 presents information regarding the factors related to RAS in the present study. Stress was identified as the most common factor, leading to recurrent ulcerations in 386 patients (54.8%). Nutritional deficiency was the second common factor related with the condition (176 patients, 25%). 53.8% of the patients with complaints of RAS were married.  Table 4 shows the attitude of the patients towards the management of RAS. 81.3% of the patients refused to get the blood investigations done. Of the 132 patients who agreed for the blood tests, 85 were tested normal, while 47 showed decreased hemoglobin, vitamin B12, serum folate and ferritin levels. Regarding the treatment, when the patients were asked about the type of treatment they prefer, 54.5% reported the use of no treatment for the condition. However, 243 patients took medications but without any consultation. Only 78 patients (11.1%) took steroids/cortisones after seeking medical consultation. 72.9% of the patients were of the opinion that there was no need to consult a dentist for this simple problem. 133 patients would however, visit the dentist only in case the situation aggravates. Only 58 patients would directly consult a dentist for RAS. Among those who did not seek any dental consultation, 68.1% were of the opinion that RAS is a simple problem, which would heal in a couple of days. 142 patients did not consider it to be a dental problem, while 65 patients opined that the treatment for these ulcers was not effective.

**Table 1:**
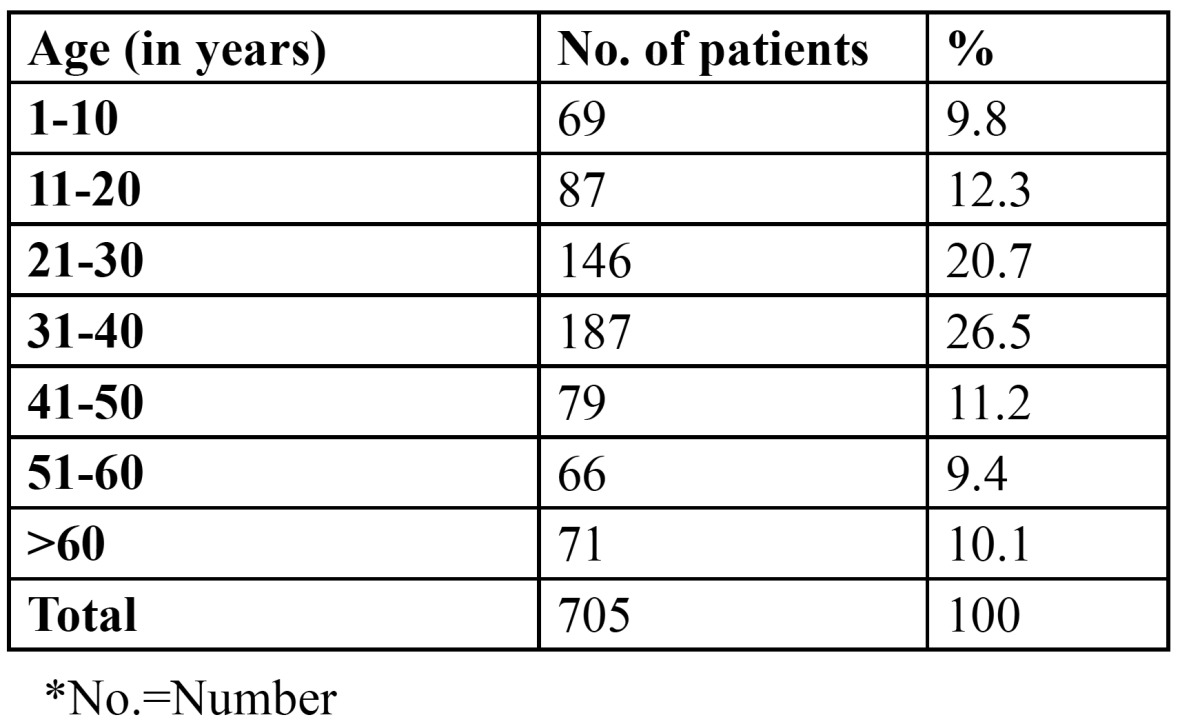
Distribution of ulcers according to age.

**Table 2:**
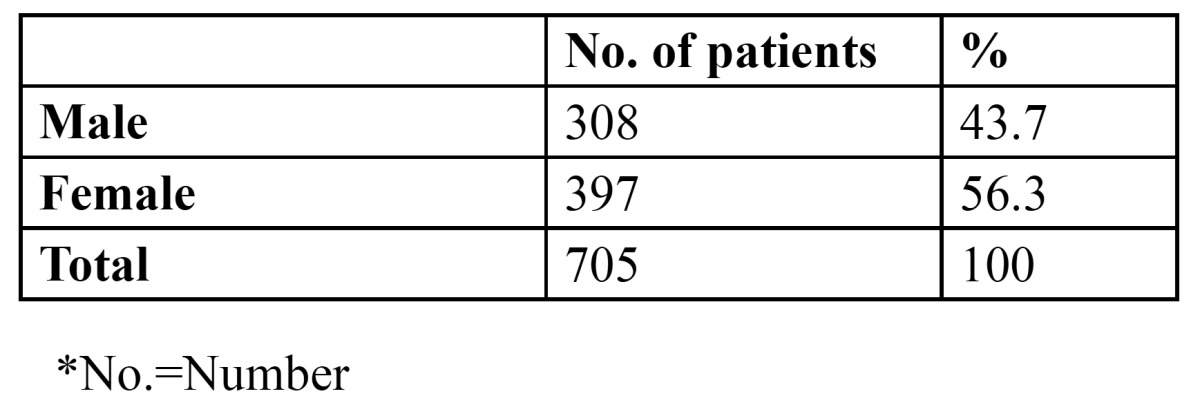
Distribution of patients according to gender.

**Table 3:**
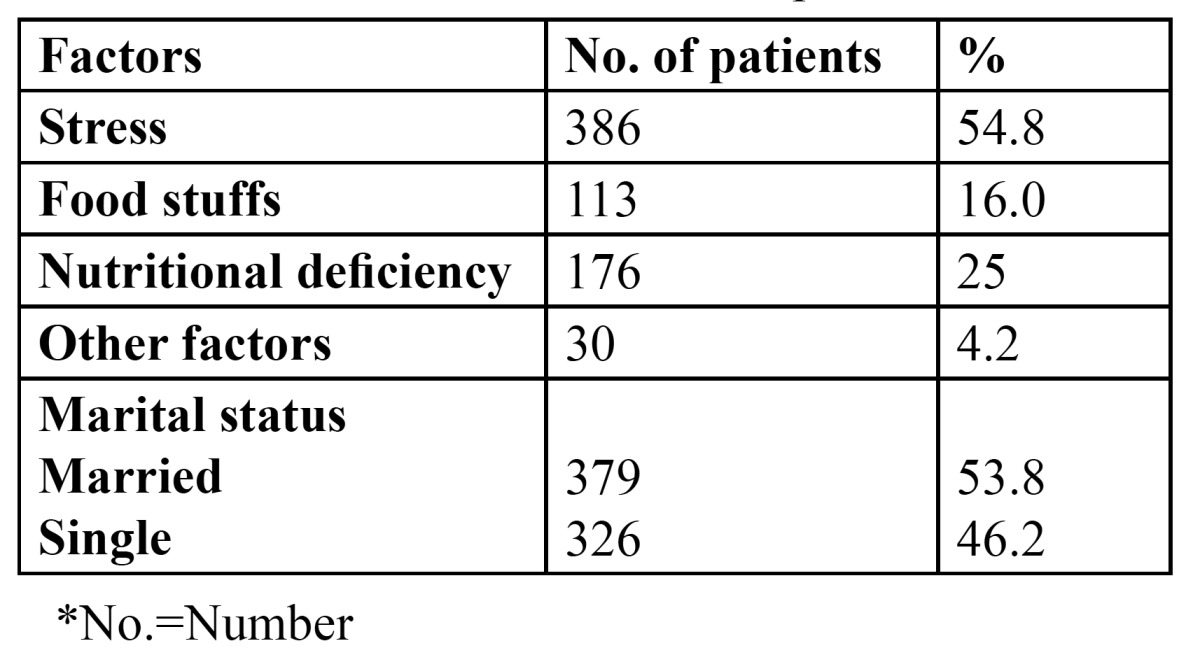
Factors related to recurrent aphthous stomatitis.

**Table 4:**
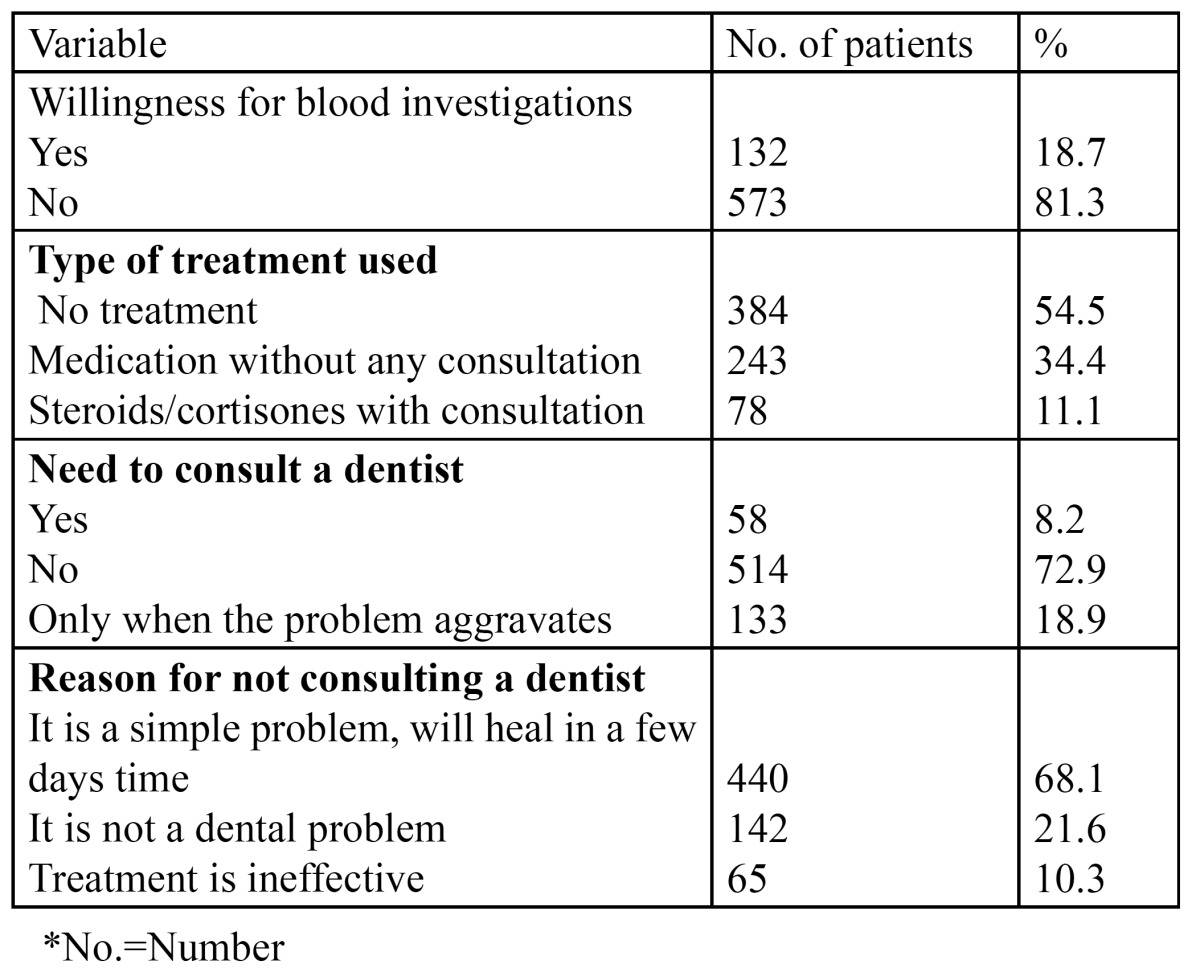
Patient’s perspective towards management of recurrent aphthous stomatitis.

## Discussion

Oral ulceration is encountered frequently in our daily practice; it causes a lot of suffering and agony for the patients throughout their life. Most ulceration are caused due to local causes such as trauma, self elicited injuries and burns. Some may result due to aphthae or malignant conditions and few may be due to underlying systemic diseases, skin disorders or autoimmune diseases (5). The present study is the first to describe the prevalence of RAS in the Indian population. No other similar study was found to address the prevalence of this group of oral mucosal lesions among the Indian population.

Recurrent aphthous stomatitis (RAS) is one of the most common painful oral mucosal conditions seen among patients. Recurrent aphthous stomatitis (RAS) is a common condition affecting the oral cavity. It is considered important, since it can be distressing and cause suffering and pain. In addition, it also interferes with normal activities by affecting eating and swallowing (7). RAS has been reported as affecting 20% of the general population at any time. The peak age of RAS onset is during childhood, with a tendency to decrease in severity and frequency with age (8). The prevalence of aphthous varies greatly among different populations and in different age groups with a range from 5-66% among different nations (9-11). The prevalence of RAS was estimated to be 40% in a sample of children in the US (1) and 25.2% in a study conducted in Iran (11). The prevalence of RAS in the present study was estimated to be 21.7%, which was in line with the study in Iran, while lower than the findings of the US population. In the present study 705 patients had recurrent aphthous stomatitis (RAS). Safadi (12) reported a much higher prevalence of 78% in the Jordanian population. Whereas, Bhatnagar at al. (13) reported a much lower prevalence of 1.53% among patients visiting a dental school in north India.

Recurrent aphthous stomatitis is characterized by recurrent bouts of one or several shallow, rounded or ovoid, painful ulcers that recur at intervals of a few days or up to 2-3 months (8). The etiology of RAS is unknown but many possible predisposing factors have been described in the literature. Much of the evidence suggests an immunologic basis for RAS. The most current etiopathogenesis indicated a cross reactivity between a streptococcal heat shock protein (hsp) and the human mitochondria hsp in the oral mucosa, which may lead to a T-cell mediated response to antigens (14). The role of environmental risk factors in the etiology of RAS is not fully understood. Deficiencies in iron, folic acid, and vitamin B12 are more common in RAS patients (15). An association has been noted between psychiatric factors and RAS but not in all studies (6). Viral or bacterial mechanisms may operate, and RAS displays some features consistent with a viral aetiology (16). RAS is also a feature of HIV/AIDS (17). In the present study stress was identified as the most common factor, followed by nutritional deficiency.

The mechanisms explaining stress as an etiological factor in RAS episodes are not fully-understood. Increased levels of salivary cortisol or of reactive oxygen species in the saliva, have been suggested as the initiator of the lesions (18,19). Due to stress, patients may begin parafunctional habits that cause traumatic injuries to the area, thus leading to an episode (18). A genetic alteration of pathways linked to stressful responses may also be involved (20). The real role of stress is still unknown but it can be probably related with the modifications that affect multiple immune system components including the distribution, proliferation and activity of lymphocytes and natural killer cells, phagocytosis, and production of cytokines and antibodies. RAS has also been linked to immune system changes, which may partially explain the role of stress in the etiology of RAS (19).

RAS has been described under 3 clinical variants as classified by Stanley (21). Minor RAS also known as Mikulicz’s aphthae or mild aphthous ulcers, is the most common variant. It constitutes about 80% of RAS. The size of the ulcers may vary from 8-10mm and is commonly seen in the non keratinized mucosal surfaces of the mouth. These ulcers show healing within 10-14 days without scarring. Major aphthous ulcer is also known as periadenitis mucosa necrotica recurrens or Sutton’s disease and affects about 10-15% of the total RAS patients (22). Major aphthous ulcers tend to be larger than minor ones and may involve the keratinised oral mucosa such as the hard palate, lips and fauces. Large ulcers may take more than 3 weeks to resolve and often leave a scar. Their clinical appearance may suggest malignancy (5). Herpetiform ulceration is characterized by recurrent crops of small, numerous ulcers; may be up to 100 in number of 1-3mm in diameter. Lesions may coalesce to form large irregular ulcers. These ulcers last for about 10-14 days and heal without scarring. Unlike herpetic ulcers, these are not preceded by vesicles and do not contain viral infected cells. These are predominantly seen in women and have a later age of onset than other variants of RAS (23).

Females (56.3%) were more frequently affected than males (43.7%) and this difference was statistically significant (p<0.005). The results were similar to the findings of Safadi (12). Females are more prone to stress and emotional situations which can affect their immune response. They seek medical examination more frequently than males. The hormonal changes during pregnancy and menstruation also play a role (14). Majority of the patients in the current study with recurrent ulcerations were of the low socioeconomic status. The high prevalence of RAS in the patients of low socioeconomic status could be attributed to poor oral hygiene, psychological factors, and lack of nutrition which may lead to deficiency of folate, ferritin, vitamins and hence low immunity to fight against various pathogens, which in turn increases the susceptibility to infective diseases. The marital status of the patients was also considered in the present study due to the stress, psychological factors, economic and social factors associated with single, married, divorced or widowed individuals.

A diagnosis of RAS mainly depends on the patients’ history, clinical examination and histopathology. Diagnostic criteria for minor RAS was proposed by Natah et al. (5). They proposed that a diagnosis of idiopathic RAS and secondary RAS (associated with systemic disease) is established when four major and one minor criteria are fulfilled. Patients with mild conditions do not require any medications. Topical steroids may be helpful to reduce the frequency and severity of the condition. Possible systemic association with RAS must be ruled out, especially in cases where there is sudden development of ulceration in adulthood (23). Blood investigations such as complete blood counts, serum folate, ferritin levels, and vitamin B12 have been recommended. However, very few patients are willing to take the investigations as seen in the present study.

Recurrent aphthous stomatitis is a very common, recurrent painful ulceration affecting the general population. The etiopathogenesis of this disease is not yet clear. Treatment strategies must be directed towards providing symptomatic relief by pain relief, increasing the duration of ulcer-free periods, and accelerating ulcer healing. Majority of the people do not consult the dentist, thinking this as a simple problem which would heal eventually in a few days, or not considering it as a dental problem. Few people do not find the treatment to be effective and are tired of visiting dentists now and then.

Understanding the prevalence and distribution of RAS among the Indian population will give an indication about the proportion of people who suffer the condition and who need dental management. Studying the prevalence of RAS gives an insight into the proportion of the population suffering from the condition and the possible related factors.

This study provides important information about the prevalence of recurrent aphthous stomatitis in India, but due to lack of similar studies in the region, no conclusion can be drawn regarding the exact prevalence. Careful examination and identification of the underlying causes will be of great help in the management of such lesions. The early and correct diagnosis of recurrent aphthous stomatitis will help the dental practitioner in providing awareness regarding the causes and management of the condition to the patient.
